# High Particulate Matter Burden of Cigarettes from the United Arab Emirates and Germany: Are There Country-Specific Differences?

**DOI:** 10.3390/ijerph17072415

**Published:** 2020-04-02

**Authors:** Markus Braun, Rawya Al-Qaysi, Doris Klingelhöfer, Ruth Müller, David A. Groneberg

**Affiliations:** 1 Institute of Occupational Medicine, Social Medicine and Environmental Medicine, Goethe University Frankfurt, Theodor-Stern-Kai 7, D-60590 Frankfurt am Main, Germany; rawya.alqaysi@yahoo.de (R.A.-Q.); klingelh@med.uni-frankfurt.de (D.K.); ruth.mueller@med.uni-frankfurt.de (R.M.); groneberg@med.uni-frankfurt.de (D.A.G.); 2 Medical Entomology, Department of Biomedical Sciences, Institute of Tropical Medicine, Nationalestraat 155, B-2000 Antwerpen, Belgium

**Keywords:** second-hand smoke, environmental tobacco smoke, indoor air pollution, tobacco control, tobacco products, declaration of tobacco ingredients

## Abstract

Although the big tobacco companies offer the same cigarette brands across countries, little is known about the potential regional differences of the particulate matter (PM) emissions of apparently equal brands. PM emissions of three cigarette brands (Marlboro Gold, Winston Red resp. Classic, Parliament Platinum resp. Night Blue) from the United Arab Emirates (UAE) and Germany were analysed. Second-hand smoke was produced in a 2.88 m^3^ measuring cabin by an automatic environmental tobacco smoke emitter. PM size fractions PM_10_, PM_2.5_, and PM_1_ were detected in real-time using laser aerosol spectrometry. Depending on the PM fraction Marlboro cigarettes from UAE showed 33%–35% higher PM amounts. Moreover, Winston cigarettes from UAE showed distinctly higher PM values (28–31%) than the German counterparts. The “lighter” Parliament from UAE emitted 3%–9% more PM than the German one. The measured mean PM_10_ values laid between 778 and 1163 µg/m^3^ (mean PM_2.5_: 777–1161 µg/m^3^; mean PM_1_: 724–1074 µg/m^3^). That means smoking in enclosed rooms causes massive PM burden. The PM emission of equal or similar tobacco products from different countries can differ distinctly. Hence, the declaration of PM emission values, besides nicotine, tar, and carbon monoxide amounts, should be obligatory worldwide. Furthermore, complete information about the ingredients and production processes of tobacco products should be provided to health officials and the public. This can help to minimise or ban substances or product designs that make smoking even more harmful, and to enhance the awareness of the risks of smoking.

## 1. Introduction

The big players of the global tobacco market offer the same cigarette brands across countries. However, the apparently identical brands may differ in production site, the origin of tobacco and, therefore, in ingredients. Hence, it is safe to assume that particulate matter (PM) emissions also vary across countries.

PM, a mixture of solid and liquid airborne particles, is classified by the United States Environmental Protection Agency (EPA) in PM_10_ (diameter ≤ 10 µm) and PM_2.5_ (diameter ≤ 2.5 µm). The EPA defines particles between 2.5 and 10 µm as coarse inhalable particles and particles ≤ 2.5 µm as fine inhalable particles [1]. The standard ISO 7708:1995 of the International Organization of Standardization (ISO) defines health-related PM size fractions by the deposition in the respiratory tract [2,3]. Such small particles can get deep into the respiratory system and even into the bloodstream. The smaller the particles, the deeper the regions they can get in, and the more adverse are the potential health effects [4]. It is reported that ultrafine particles (UFP; <100 nm) can reach the head region and the brain probably via the olfactory bulb [5]. PM is associate with a variety of serious health effects, especially heart and respiratory diseases, which can prove to be fatal [6]. The International Agency for Research on Cancer (IARC) of the World Health Organization (WHO) classifies PM as carcinogenic to humans [7]. In general, the higher the exposure to PM, the higher are morbidity and mortality [8]. Lim et al. [9] estimated in a comparative risk assessment for the year 2010 about 3.1 million cases of death caused by PM worldwide. The WHO even stated that 4.2 million cases of premature death have been caused by ambient air pollution and 3.8 million cases have been caused by indoor air pollution [10].

There are many sources of PM both indoor and outdoor [11]. Second-hand smoke (SHS), also known as environmental tobacco smoke, is mainly composed of smoke from the smouldering tobacco product and, with a minor part, of exhaled mainstream smoke from the smoker. It is the main indoor source of PM in households of smokers [12]. High levels of particle-bound polycyclic aromatic hydrocarbons (PAHs), whereof several are carcinogenic, were found in smokers’ homes [13].

The present investigation aimed to ascertain if any country-specific differences of PM emissions by tobacco smoke are detectable. Two apparently identical pairs of two cigarette brands (Marlboro, Winston) and additionally a “light” and a “normal” type of the same brand (Parliament) sold in the United Arab Emirates (UAE) and Germany (GER) were examined regarding PM in SHS. This seems to be reasonable because previous studies showed a wide range of PM levels within different tobacco products [14,15,16,17]. The high-income countries UAE and Germany were chosen due to their similarities in a socioeconomic context [18].

Nevertheless, the UAE and Germany differ regarding tobacco consumption and its consequences in various ways. As maintained by *The Tobacco Atlas* of the *American Cancer Society* [19], in 2015, more than 1,342,000 adult people (15+ years old) in the UAE used a tobacco product each day, meaning 21.6% of men and 1.9% of women of its people. In Germany, the number was more than 15,341,000 adult people (25.1% of men and 17.1% of women of its people), who use tobacco products daily. Thus, the prevalence of female smoking is in the UAE is much lower than in Germany. In 2016, deaths caused by tobacco were, in the UAE, 2900 of its people lower than the average in very-high-HDI (Human Development Index) countries (male 12.84%, female 6.05% of total number of deaths). In Germany, the numbers are higher than on average in very-high-HDI countries given that more than 124,800 people died owing to tobacco consumption (male 18.42% and female 8.93% of the total number of deaths). According to *The Tobacco Atlas*, smoke-free policies are stronger in the UAE than in Germany, especially in healthcare and educational facilities. Additionally, bans on advertising are more extensive in the UAE. For example, in Germany, billboard and outdoor advertising, and advertising at selling points are still allowed.

## 2. Materials and Methods

### 2.1. Tobacco Products

The 3R4F reference cigarette developed by the *Kentucky Tobacco Research and Development Center* of the University of Kentucky, USA, [20] served as the standard of comparison for each three cigarette brands from the UAE and Germany. The UAE cigarettes were bought at an International Airport of Dubai and are as follows: Marlboro Gold [21], Winston Red [22], and Parliament Platinum [21]. The cigarettes from Germany were bought at an International Airport of Frankfurt and are as follows: Marlboro Gold [21,23], Winston Classic [22,23], and Parliament Night Blue [21,23]. Marlboro Gold and the two Parliament brands were produced by *Philip Morris GmbH* in Germany for the market of both countries. For the German market, Winston Classic cigarettes were manufactured by *Japan Tobacco International* (JTI) in Germany, whereas Winston Red from the UAE were produced by JTI in Switzerland. Table 1 shows some characteristics of all investigated tobacco products, whereby information on the manufacturer and amounts of nicotine, tar, and carbon monoxide were taken from the respective cigarette pack. The sizes and weights are given as measured means of five randomly chosen cigarettes of each brand.

### 2.2. Automatic Environmental Tobacco Smoke Emitter

To generate SHS reproducibly, an Automatic Environmental Tobacco Smoke Emitter (AETSE) was used that was developed for the Tobacco Smoke Particles and Indoor Air Quality (ToPIQ) studies [14,24] and constructed by *Schimpf-Ing.* Trondheim, Norway [25]. The programmable microprocessor-controlled AETSE serves as a smoke pump and imitates the smoker. A 200-mL glass syringe, connected via a polyamide tube with the mouthpiece of the tobacco product, is moved by a stepper motor and take puffs of the tobacco product. After each puff, the smoke is pressed valve-controlled into the measuring chamber. The chamber with an internal volume of 2.88 m^3^ is closed during the smoke processes, avoiding the exposure of any person to tobacco smoke.

### 2.3. Smoking Protocol

The number of cigarettes analysed was *n* = 20 for each tested type. All investigated cigarettes were smoked following the same modified protocol according to the ToPIQ studies [14,24]. The setting of the AETSE for the combustion phase, where each cigarette was lighted and smoked, was as follows: 40 mL puff volume; 13 mL/s puff flow rate; 2 puffs/min; duration 3 s/puff; two ignition puffs followed by 7 regular puffs. This corresponds to a 4 min 22 s combustion phase. After that, each cigarette was extinguished followed by the 5 min post-combustion phase. Then, the air in the measuring chamber was cleaned by ventilating with an industrial radial fan for at least 5 min. Subsequently, a new cycle began with a blank measurement in the pre-ignition phase.

### 2.4. Laser Aerosol Spectrometer

The applied Laser Aerosol Spectrometer (LAS) and Dust Monitor Model 1.109 of Grimm Aerosol Technik GmbH & Co KG (Ainring, Germany) [26] can detect airborne particles every six seconds in real-time. The measuring size ranges from 0.25 to 32 µm. The suction point of the LAS is placed 35 cm beside of the tobacco product at the same height. It is necessary to dilute the sample air pre-analytically using compressed air at a ratio of 1:10 to avoid blockage of the LAS measurement chamber. The dilution ratio was taken into account at the following data processing. Among others, the measured data can be represented in terms of occupational health according to the European Standard EN 481 [27], inhalable, thoracic, and alveolic in µg/m^3^, and according to U.S. EPA as dust mass fractions PM_10_, PM_2.5_, [1] and additionally PM_1_ in µg/m^3^.

### 2.5. Data Processing

For all investigated cigarettes, the mean concentrations (C_mean_) and the area under the concentration-time curve (AUC), meaning the total PM exposition, were calculated for data received during the 4 min 22 s of the combustion phase. All C_mean_ and AUC values were tested for Gaussian distribution that all data passed. Subsequently, a one-way analysis of variance (ANOVA) test and Tukey’s multiple comparisons test were applied to show the differences between different types of cigarettes among themselves. Finally, a two-way ANOVA test, including Sidak’s multiple comparisons test, was done to examine the country-specific differences. The level of significance was set on *p* = 0.05. All statistical tests were done with GraphPad Prism version 8 (San Diego, CA, USA) software.

## 3. Results

Table 2 listed the C_mean_ and AUC values of all investigated cigarette brands. Looking at the mean PM measurement results, both Marlboro and Winston cigarettes from UAE show significantly higher values than the German counterparts. The C_mean_ PM_1_ value of Marlboro UAE is 1074 µg/m^3^ 35% higher than the mean value of the Marlboro GER (795 µg/m^3^). The Winston UAE show a 28% higher C_mean_ PM_1_ value (929 µg/m^3^) than those from Germany (724 µg/m^3^). The C_mean_ value of 997 µg/m^3^ for PM_1_ of the Parliament UAE cigarettes is only a little higher (9%) than that of the German (917 µg/m^3^). The UAE cigarettes of all three brands also show higher PM_10_ and PM_2.5_ C_mean_ values. Accordingly, Marlboro UAE shows 33% higher PM_10_ and PM_2.5_ mean values, and Winston UAE shows 31% and 30% higher values, respectively, than the German counterparts. Both Parliament brands show very similar PM_10_ and PM_2.5_ measurement results with only 3% higher values than the UAE type. Regarding the AUC values, similar findings emerged.

**Figure 1:**
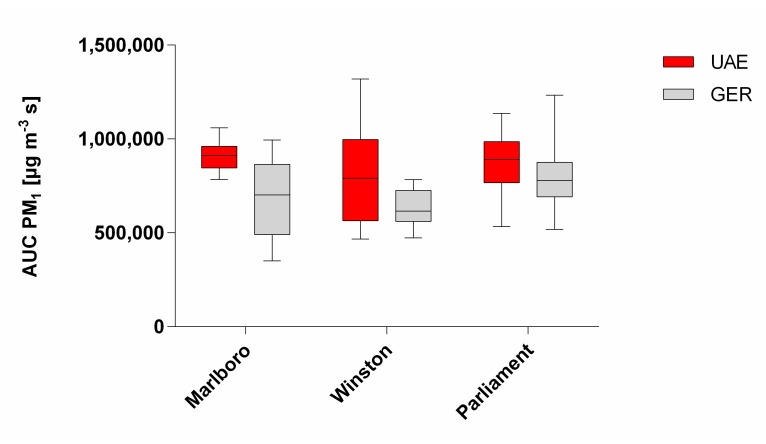
Comparative boxplot (min to max whiskers) of the area under the curve (AUC) PM_1_ of country-specific brands. The results are based on a statistical two-way analysis of variance (ANOVA) test. UAE = United Arab Emirates. GER = Germany.

The two-way ANOVA test of the investigated pairs of cigarette brands revealed that the origin of the tobacco product has a higher influence on PM emissions than the brand (Figure 1). A pairwise comparison regarding the p values of the brands from UAE and Germany are shown in Table 3. The country-specific significance for PM_1_ is *p* < 0.0001 (brand: *p* = 0.0114). An interaction between brand and country is not significant (*p* = 0.123). Similar results pertain for PM_2.5_ (country: *p* = 0.0005; brand: *p* = 0.0097; interaction: *p* = 0.0907) and PM_10_ (country: *p* = 0.0006; brand: *p* = 0.0097; interaction: *p* = 0.0892).

The C_mean_ and AUC values of Marlboro UAE do not differ significantly from the values of the reference cigarette. A little lower, but not significantly, are the values of Parliament UAE (−4.2% for C_mean_ PM_10_), Parliament GER (−6.6% for C_mean_ PM_10_) and Winston UAE (−1.4% for C_mean_ PM_10_). Significantly lower compared to the reference cigarette are the values of the Marlboro GER (−23.8% for C_mean_ PM_10_; *p* = 0.0139) and the Winston GER (−32.2% for C_mean_ PM_10_; *p* = 0.0002).

**Figure 2:**
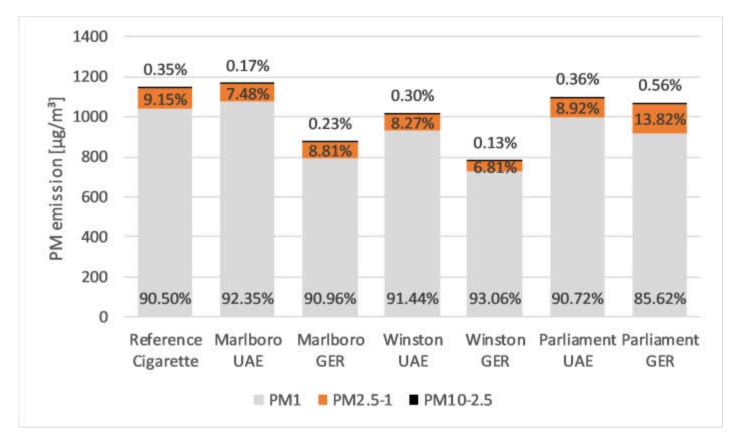
Distribution pattern of PM_10–2.5_, PM_2.5–1_, and PM_1_ in aerosol generated by the combustion of all investigated cigarettes. UAE = United Arab Emirates. GER = Germany.

Regarding the distribution pattern of the particles PM_10_, PM_2.5_, and PM_1_ (Figure 2), it was ascertained for all investigated brands that the main part is represented by the PM_1_ fraction with a percentage from about 85% to 93%. The proportion of PM_10_ is far below 1% for all brands.

## 4. Discussion

The measured C_mean_ PM_2.5_ values were between 777 µg/m^3^ (Winston GER) and 1161 µg/m^3^ (Marlboro UAE). By way of comparison, the WHO limit value (24-h mean) for ambient air PM_2.5_ concentration is 25 µg/m^3^ [28]. In addition to this, the WHO air quality guideline pointed out that there is no safe level below which no risk for health exists by PM exposure [28]. This means smoking in closed rooms causes PM burden with strong exceedance of the WHO 24-h mean limit value accompanied by a considerably increased risk for adverse health effects. It would take hours until the PM values decrease to a level below the limit. Semple and Latif [29] ascertained in an analysis of PM_2.5_ amounts in SHS in households of smokers a PM half-life (50% PM decline) of about one hour.

The scenario of our study in the measuring cabin with an indoor volume of 2.88 m^3^ is comparable to the situation in a compact car with closed windows and turned off ventilation. This car class has a passenger and cargo volume of 2.832 to 3.087 m^3^ according to U.S. EPA [30] and, hence, a similar internal volume. Considering the very high PM burden caused by smoking in such small closed rooms and the associated health risks, a global ban on smoking in cars is to be in order, especially as children as car passengers are often affected.

Both tested Marlboro cigarettes, one type produced for the UAE sales market, and the other for the German market, were manufactured in Germany and had identical nicotine, tar, and carbon monoxide amounts. Nevertheless, our findings show that the UAE type emitted significantly more PM. The second investigated brand, Winston, from the UAE with lower amounts of nicotine, tar, and carbon monoxide emitted more PM too. Parliament GER and Parliament UAE showed very similar PM values, although the UAE type is a “lighter” version. Braun et al. [31] ascertained in an investigation of four cigarette types of one specific brand, all manufactured for the German market, that the lower the amounts of nicotine, tar, and carbon monoxide, the less PM is in the SHS. However, the results of our study suggest that smoking cigarettes from the UAE leads to a higher PM burden than smoking German cigarettes, especially at a similar tobacco strength. Looking at the six investigated brands, the high statistical significance of the country-specific differences in contrast to the obvious lower significance of the influence of the brand on PM emissions has to be emphasised.

The reasons for the varying PM emission values are not clear. However, it can be assumed that tobacco companies use country-specific tobacco blends and/or filters and/or cigarette paper for the respective tobacco market. A different composition of additives, different tobacco blend, or a combination of both are plausible reasons for our findings. The tobacco use in both countries differs in the choice of specific tobacco products as well as sociologically. First, it is worth mentioning that in the UAE the proportion of female smokers is very low, but the male smoking rates are much higher and comparable to those of Germany. According to the *WHO Report on the Global Tobacco Epidemic, 2019* [32], the prevalence in the UAE of the current smoking of any tobacco product among persons aged ≥ 15 years is 14.8% (male 29.1%, female 0.6%), and of cigarette smoking 11.4% (male 22.5%, female 0.3%). The respective data for Germany related to smoking any tobacco product is 28.4% (male 30.4%, female 26.3%) and of cigarette smoking 26.3% (male 28.1%, female 26.3%). In the Middle East, traditional and centuries-old tobacco use with a water pipe (i.e., hookah, shisha, narghile) is becoming more and more popular worldwide. This trend started in the 1990s among the Arab youth [33]. Al-Houqani et al. [34] reported in a large cross-sectional survey from 2008 to 2010 among 170,430 UAE nationals aged ≥18 years that the prevalence of smoking a water pipe was in total 0.76% (male 1.67%). This was trumped by the prevalence of smoking cigarettes (in total 8.55%, male 18.77%) and smoking Midwakh (in total 1.66%, male 3.64%). Midwakh, traditionally smoked by Bedouins and sailors of the Gulf States, is a small pipe for smoking dry tobacco (Dokha), often flavoured with spices and herbs [34]. In Germany, there exists no data about midwakh smoking, but the use of a midwakh might certainly be unusual. The prevalence of the current smoking of water pipes among German persons aged ≥15 years is declared by the *Special Eurobarometer 385* survey with 5% [35]. Unfortunately, all this data is based on surveys and are, therefore, not consistent. In 2018, Al-Houqani et al. [36] compared in a pilot study the self-reported tobacco use of 399 UAE nationals with the validation of the smoking status via cotinine testing in urine samples. They found that 42% of men and 9% of women were positive for cotinine versus the self-reported tobacco use of 36% men and 3% women. This indicates that especially women in the UAE often conceal the use of tobacco. Nevertheless, all this elucidate significant differences in the use of tobacco between UAE and Germany.

Tobacco companies declare the exact composition, the “recipe” of the tobacco product, as “highly valuable trade secrets” [21,22,37]. O’Connor et al. [38] concluded in a comparison of cigarettes from ten different countries (low-, middle- and high-income) that the higher the country’s economic level, the lower the emission levels due to a higher engineering of cigarette design and filters. On the other side, Song et al. [39] demanded a general ban on ventilated filters, which can be referred to as “highly engineered”. Ventilated filters lead to a lower combustion temperature and the subsequent more incomplete combustion that increases the toxicity of tobacco smoke. Moreover, the smoker is tempted to inhale more intensively while smoking tobacco products with ventilated filters.

Previous study results regarding additives are inconsistent. On the one hand, Rustemeier et al. [40] stated in 2002 that additives in cigarettes increase PM levels in a range from 13% to 28%. Braun et al. [17] found higher PM burden by cigarettes with additives in comparison to those of the same brand without additives too. An analysis of the formerly secret documents of the tobacco industry released in 2011 by Wertz et al. [41], ascertained post hoc protocol alterations in four peer-reviewed publications conducted by Philip Morris. In this way, the initial statistical findings that additives lead to an increase in toxicity as well as PM concentrations in tobacco smoke should be obscured. On the other hand, Wasel et al. [15] found no statistically significant increase of PM amount in cigarette smoke caused by additives in an investigation of four L&M tobacco products, one type without additives. Gaworski et al. [42] and Gerharz et al. [43] examined cigarettes with the additive menthol. They could not prove an increase in neither toxicity nor PM burden caused by menthol. 

A limitation of this study was that the used LAS Grimm Model 1.109 detects airborne particles in a size range of 0.25 to 32 µm, but not below. The detected particles of all brands were mainly composed by PM_1_ (85%–93%) depending on the brand. Only 0.13% to 0.56% were sized between 2.5 and 10 µm (Figure 2). This implies that a part of submicronic particles (<1 µm) and UFPs (<100 nm) are not included in this analysis. Several studies reported different particle sizes (e.g., 0.1–1 µm or 0.02–2 µm) and mean diameters (e.g., 0.1, 0.18, or 0.5 µm) of tobacco smoke [44,45,46,47,48,49]. Hence, it is safe to assume that the measured data of this study are slightly to low. Nevertheless, the applied LAS is able to detect most of the PM in SHS. However, since the adverse health effects of UFPs have come more and more into focus [50], an expansion of the measurement system would be useful.

The reference methods for measuring PM stated by the European Committee for Standardisation (see: standard EN 12341) [51] and the US EPA (see: Federal Reference Methods, FRM) [52] are mostly gravimetric methods. After a 24-h sample collection of PM, the sample filters will be weighed out to determine the mass concentrations of PM_10_ and PM_2.5_ without declaration of PM_1_. The LAS Grimm Model 1.109, which was used in this study, measures PM in real-time via light scattering like the Grimm Model EDM 180 or the Tapered Element Oscillating Microbalance (TEOM) Monitor, which are both FRMs too [52]. A study from 2009 showed very similar PM measuring data of the gravimetric methods, the TEOM Monitor, Grimm Model EDM 180, and the Grimm Model 1.109 [53]. Fromme et al. [54] noticed that PM values measured by LAS are lower than those of the gravimetric methods but with a very high correlation of the data rank order. Thus, it is essential not to change the measuring method or device during the investigation. Hence, the measuring data of the used LAS can be considered as valid. Besides, it has to be emphasised that the applied LAS enables us to investigate each single tobacco product in real-time.

There are several protocols for smoking regimes, but no “gold standard”. Here, the standard of the *International Organization of Standardization* ISO/TR 17219:2013 for machine smoking of cigarettes [55] or the *Standard Operating Procedure for Intense Smoking of Cigarettes* provided by the WHO [56] shall be mentioned. The protocol underlying the current paper differs from these smoking regimes, but the provided results are likewise reliable and reproducible. The here applied AETSE produces SHS without exposing any person to tobacco smoke and concomitant health risks. The exact imitation of human smoking behaviour and SHS is not possible as true smoking humidifies the inhaled mainstream smoke in the respiratory tract. This leads to hygroscopic growth, and afterwards, the particles of the exhaled smoke are about 1.5-fold larger [57]. As SHS is a composite of about 85% side-stream smoke and only 15% mainstream smoke [58], the AETSE generates tobacco smoke that is very similar to SHS.

This study, much like to previous ToPIQ studies, focused on the comparison of the investigated tobacco products with a reference cigarette [14,15,16,17,24,31,43,59]. Especially for comparison with guideline values or other investigations, the absolute data are stated.

## 5. Conclusions

Especially in small settings and non-aerated rooms, smoking leads to massive PM burden. Even though the manufacturing locations are equal, the PM emissions of similar tobacco products from various countries can differ significantly. Tobacco products are manufactured for respective tobacco markets with different legal norms. Therefore, a global and consistent obligation to declare the amounts of nicotine, tar, carbon monoxide and PM amounts is useful and necessary. Additionally, it should be obligatory for tobacco companies to fully inform health officials, the public, and projects, e.g., DiMoPEx (*Diagnosis, Monitoring and Prevention of Exposure-related Non-Communicable Diseases*) by the *European Cooperation in Science and Technology* [60,61], about ingredients and production design of their tobacco products. This information can help to determine all characteristics of tobacco products (e.g., tobacco blend, additives or filter design) that make smoking even more harmful. Additionally, this knowledge would enable the regulation by law and the purposeful information of the public. Stronger legal regulations and public awareness will help to reduce the distribution of tobacco products worldwide to protect the human health.

The findings of this study need further investigations on comparisons of international tobacco products. Hence, studies on country-specific tobacco products will follow.

## Figures and Tables

**Table 1:** Features of the tested cigarette brands. The cigarette dimensions and weights are the mean values of five randomized chosen cigarettes of each brand. UAE = United Arab Emirates. GER = Germany.

Cigarette Brand (Country of Origin)	Reference Cigarette 3R4F (USA)	Marlboro Gold (UAE)	Marlboro Gold (GER)	Winston Red (UAE)	Winston Classic (GER)	Parliament Platinum (UAE)	Parliament Night Blue (GER)
Manufacturer (Production location)	KTRDC Univ. of Kentucky (USA)	Philip Morris GmbH (GER)	Philip Morris GmbH (GER)	Japan Tobacco International (Switzerland)	Japan Tobacco International (GER)	Philip Morris GmbH (GER)	Philip Morris GmbH (GER)
Nicotine [mg]	0.73	0.5	0.5	0.6	0.8	0.1	0.8
Tar [mg]	9.4	6	6	7	10	1	10
Carbon Monoxide [mg]	12	7	7	7	10	1	10
Total Length [mm]	84	83	83	84	83	83	98
Total Weight [mg]	988	807	797	807	767	807	1045
Tobacco Weight [mg]	775	599	580	653	642	600	755
Filter Length [mm]	27	27	27	21	21	22	22
Filter Diameter [mm]	8	8	8	8	8	8	8

**Table 2:** Mean concentrations (C_mean_ PM_10_, PM_2.5_ and PM_1_) and Area Under the Curve (AUC PM_10_, PM_2.5_ and PM_1_) with standard deviation of all tested tobacco products. UAE = United Arab Emirates. GER = Germany.

Cigarette Brand (Country)	Reference Cigarette 3R4F (USA)	Marlboro Gold (UAE)	Marlboro Gold (GER)	Winston Red (UAE)	Winston Classic (GER)	Parliament Platinum (UAE)	Parliament Night Blue (GER)
C_mean_ PM_10_ [µg/m^3^]	1147 ± 131	1163 ± 165	874 ± 284	1016 ± 367	778 ± 140	1099 ± 230	1071 ± 332
C_mean_ PM_2.5_ [µg/m^3^]	1143 ± 127	1161 ± 163	872 ± 282	1013 ± 365	777 ± 139	1095 ± 228	1065 ± 324
C_mean_ PM_1_ [µg/m^3^]	1038 ± 83	1074 ± 119	795 ± 228	929 ± 301	724 ± 109	997 ± 183	917 ± 184
AUC PM_10_ [µg/m^3^ s]	993,700 ± 114,216	982,886 ± 108,338	756,938 ± 245,919	879,302 ± 318,628	673,982 ± 121,369	945,533 ± 198,399	927,093 ± 287,701
AUC PM_2.5_ [µg/m^3^ s]	989,999 ± 110,121	981,310 ± 107,531	755,173 ± 244,487	877,279 ± 317,060	672,827 ± 120,476	943,460 ± 197,337	922,547 ± 280,833
AUC PM_1_ [µg/m^3^ s]	898,660 ± 72,073	913,681 ± 83,482	688,275 ± 197,675	803,915 ± 261,703	626,772 ± 94,302	863,535 ± 158909	793,771 ± 159,949

**Table 3:** *p*-values of statistical Sidak’s multiple comparisons test of C_mean_ (PM_10_, PM_2.5_, PM_1_) for the three cigarette brand pairs from the UAE and Germany.

	Marlboro	Winston	Parliament
PM_10_	**0.0026**	**0.0194**	0.9838
PM_2.5_	**0.0023**	**0.0185**	0.9791
PM_1_	**< 0.0001**	**0.0052**	0.5171

Statistical significance regarding the origin of tobacco product is highlighted by a bold font type. The significance level was set on *p* = 0.05.
